# Development and assessment of a natural language processing model to identify residential instability in electronic health records’ unstructured data: a comparison of 3 integrated healthcare delivery systems

**DOI:** 10.1093/jamiaopen/ooac006

**Published:** 2022-02-16

**Authors:** Elham Hatef, Masoud Rouhizadeh, Claudia Nau, Fagen Xie, Christopher Rouillard, Mahmoud Abu-Nasser, Ariadna Padilla, Lindsay Joe Lyons, Hadi Kharrazi, Jonathan P Weiner, Douglas Roblin

**Affiliations:** 1 Center for Population Health Information Technology, Department of Health Policy and Management, Johns Hopkins Bloomberg School of Public Health, Baltimore, Maryland, USA; 2 Institute for Clinical and Translational Research, Johns Hopkins Medical Institute, Baltimore, Maryland, USA; 3 Kaiser Permanente Southern Caifornia, Pasadena, California, USA; 4 Kaiser Permanente Mid-Atlantic States, Rockville, Maryland, USA; 5 Department of Medicine Division of Health Sciences Informatics, Johns Hopkins School of Medicine, Baltimore, Maryland, USA

**Keywords:** natural language processing, electronic health record, homelessness, housing insecurity, social determinants of health

## Abstract

**Objective:**

To evaluate whether a natural language processing (NLP) algorithm could be adapted to extract, with acceptable validity, markers of residential instability (ie, homelessness and housing insecurity) from electronic health records (EHRs) of 3 healthcare systems.

**Materials and methods:**

We included patients 18 years and older who received care at 1 of 3 healthcare systems from 2016 through 2020 and had at least 1 free-text note in the EHR during this period. We conducted the study independently; the NLP algorithm logic and method of validity assessment were identical across sites. The approach to the development of the gold standard for assessment of validity differed across sites. Using the EntityRuler module of spaCy 2.3 Python toolkit, we created a rule-based NLP system made up of expert-developed patterns indicating residential instability at the lead site and enriched the NLP system using insight gained from its application at the other 2 sites. We adapted the algorithm at each site then validated the algorithm using a split-sample approach. We assessed the performance of the algorithm by measures of positive predictive value (precision), sensitivity (recall), and specificity.

**Results:**

The NLP algorithm performed with moderate precision (0.45, 0.73, and 1.0) at 3 sites. The sensitivity and specificity of the NLP algorithm varied across 3 sites (sensitivity: 0.68, 0.85, and 0.96; specificity: 0.69, 0.89, and 1.0).

**Discussion:**

The performance of this NLP algorithm to identify residential instability in 3 different healthcare systems suggests the algorithm is generally valid and applicable in other healthcare systems with similar EHRs.

**Conclusion:**

The NLP approach developed in this project is adaptable and can be modified to extract types of social needs other than residential instability from EHRs across different healthcare systems.

## Lay Summary

We evaluated the performance of a natural language processing (NLP) algorithm to extract markers of residential instability (ie, homelessness and housing insecurity) from electronic health records (EHRs) of 3 healthcare systems. We included patients 18 years and older who received care at 1 of 3 healthcare systems from 2016 through 2020 and had at least 1 free-text note in the EHR during this period. We conducted the study independently; the NLP algorithm logic and method of validity assessment were identical across sites. The approach to the development of the gold standard for assessment of validity differed across sites. We created a rule-based NLP system made up of expert-developed patterns indicating residential instability at the lead site and enriched the NLP system using insight gained from its application at the other 2 sites. We assessed the performance of the algorithm by measures of positive predictive value (PPV), sensitivity, and specificity. The NLP algorithm performed with moderate PPV (0.45, 0.73, and 1.0), sensitivity (0.68, 0.85, and 0.96), and specificity (0.69, 0.89, and 1.0) across the 3 sites. Our findings suggest that this approach to extracting information on social needs from the free-text EHR notes is generally applicable in other healthcare systems with similar EHRs.

## INTRODUCTION

### Background and significance

Successful healthcare delivery goes beyond addressing patients’ medical conditions and involves addressing patients’ social needs and social determinants of health (SDOH).^1–5^ Social needs include immediate individual level needs, such as housing instability and inadequate nutrition.^6–13^ SDOH includes circumstances at the community level, such as unsafe neighborhoods and living in a food desert.^14^^,^^15^ Social needs and SDOH challenges contribute to increased healthcare costs and utilization and decreased life expectancy.^16^^,^^17^

While the International Classification of Diseases 10th revision (ICD-10) coding system accommodates documentation of social risks and needs, recording of these nonclinical issues in the electronic health records (EHR) may rarely occur as their recognition as part of the etiology of a disease is poorly understood and addressing social risks and needs is not typically reimbursable. The completeness or validity of structured ICD codes for identification of social risks and needs, therefore, may be inadequate for managing population health or targeting high-risk patients for intervention.^18^^,^^19^

Despite the lack of coding in the EHRs, social risks and needs may be discussed with healthcare providers during visits and recorded in EHRs as free-text notes. These notes might provide a more complete or accurate accounting of such needs. However, traditional approaches for the review and abstraction of patient information from medical record notes are laborious, expensive, and slow.

Recent developments in text mining and natural language processing (NLP) of digitized text allow for reliable, low-cost, and rapid extraction of information from EHRs.^13^^,^^19–24^ Developing NLP algorithms that could function in different healthcare systems would improve the application of such methods in extracting social needs from the EHR’s free text.

### Objectives

We conducted a pilot study to evaluate whether an NLP algorithm could extract valid measures of social needs from Epic-based EHRs in 3 different healthcare systems: Johns Hopkins Health System (JHHS), Kaiser Permanente Mid-Atlantic States (KPMAS), and KP Southern California (KPSC).

## MATERIALS AND METHODS

### Study design

We conducted this study independently, in a parallel and coordinated framework across the healthcare systems. We included patients 18 years of age and older who received care at JHHS, KPMAS, and KPSC from 2016 through 2020 and had at least 1 free-text note in their EHR during the study period. The focus of our study was residential instability (ie, homelessness and housing insecurity). Supplementary Table S1 defines residential instability used across the study sites. The study protocol was reviewed and approved by the Institutional Review Board at each study site.

### Developing training and validation data sets

Each study site developed training and validation data sets according to their data availability.

The JHHS site assessed over 30 EHR questionnaires and flowsheets, available in the EHR structured data, addressing residential instability and identified 5 relevant ones. We identified 1786 patients with a positive response and 45 654 patients with a negative response to residential instability questions. We included the 1786 patients with a positive response in the training data set and randomly selected 1786 patients with a negative response to the same questions to add to the data set. We used patient responses to generate a binary label of a patient’s residential instability (Supplementary Table S2). We assigned a positive label (1) to patients with a response indicating an unmet housing need and a negative label (0) to patients with a response indicating no current housing need. We extracted provider notes occurring ±30 days of the questionnaire’s date and linked them to each questionnaire result. If multiple questionnaires were completed within 60 days of each other, we assigned the overlapping provider notes to the latest questionnaire date. If any text of the selected questionnaires were identified in the provider’s notes (some providers may copy/paste a questionnaire into a note), we excluded the text to assure it would not impact the performance of our NLP algorithms. We did not have any limitations in selecting the provider notes and only excluded lab results and radiology and pathology reports. We randomly selected 50% of the sample to develop the training data set and reserved the remaining subset for evaluation of model over-fitting in a hold-out validation data set.

The KPMAS site extracted the Your Current Life Situation (YCLS) survey data from the EHR, administered in written form, or electronically via the KP.org website. We identified a total of 40 372 YCLS survey responses completed by 25 727 KPMAS adult members. We used patient YCLS survey responses to generate a binary label of a patient’s needs related to residential instability (Supplementary Table S2). We assigned a positive label (1) to patients with a survey response indicating an unmet housing need and a negative label (0) to patients with a survey response indicating no current housing need. We extracted providers' notes occurring ±30 days of the survey’s date and linked them to each survey result. If multiple surveys occurred within 60 days of each other, we assigned the overlapping provider notes to the latest survey’s date. We limited the provider notes to case management, complex care program, family practice, internal medicine, psychotherapy, and utilization management departments. We randomly selected 80% of the total sample to develop the training data set. We grouped provider notes by their associated YCLS survey before the random data split, ensuring that all notes linked to 1 survey remained together following the data split. This split reserved 20% of the study sample for evaluation of model over-fitting in a hold-out validation data set.

The KPSC site extracted data on individuals with an emergency department visit or hospitalization record during the study period. We randomly selected 150 patients with either a documented homeless status in a structured field in which providers can indicate homelessness or an ICD-10 diagnosis code of homelessness/housing issues. Those with a housing issue were assigned a positive residential instability label (1) and patients without documented homeless status and relevant ICD-10 codes were assigned a negative label (0). We then extracted providers’ notes occurring ±14 days of the ED or hospitalization encounters. We excluded notes such as discharge instructional notes as they lacked any specific details of the patient’s social conditions and needs. A team of trained and experienced researchers conducted a full medical record review, performed manual annotation independently to determine the residential instability status for the selected study sample, and documented the reasons for assigning each candidate to the positive and negative social labels as well as supporting evidence for each assignment. The study team reviewed and resolved the discrepancies between residential instability labels generated from ICD-10 codes and the results of the manual annotation by reviewing all information available in the medical record.

We then randomly split the total sample into 5 subsets, each set containing 30 patients from the positive and 30 patients from the negative residential instability groups. We used 4 of the subsets for the iterative adaption of the NLP algorithm. That is, we used the NLP algorithm developed by the JHHS team to identify residential instability among the first set of 30 patients from the positive and 30 patients from the negative residential instability groups. We compared those with the findings from the annotated chart review and modified the NLP algorithm accordingly. We then repeated this procedure 3 more times. Finally, we used the fifth set of 30 positive and 30 negative patients for the final evaluation of model over-fitting as a hold-out validation data set.

### Feature development

We used both manual lexicon curation and semiautomated lexicon creation for feature development.^24^ To develop hand-crafted linguistic patterns, a team of subject matter experts at JHHS reviewed ICD-10, Current Procedural Terminology (CPT), Logical Observation Identifiers Names and Codes (LOINC) codes, and Systematized Nomenclature of Medicine—Clinical Terms (SNOMED CT) terminologies to identify codes and phrases related to residential instability.^25^^,^^26^

The expert team also reviewed the description of residential instability in public health surveys and instruments such as the Protocol for Responding to and Assessing Patients’ Assets, Risks, and Experiences (PRAPARE) and similar surveys.^27–30^ Additionally, our expert team reviewed phrases derived from a literature review and the results of a manual annotation process from a past study.^20^^,^^21^ To finalize the linguistic patterns, the expert team developed a comprehensive list of all available codes, specific content areas, and phrases for residential instability. Then matched them across different coding systems and developed several phrases and synonyms to describe each content area. Supplementary Table S1 presents sample phrases for residential instability. These phrases were then converted to 47 unique patterns in spaCy. The team did not assess temporality in the occurrence of residential instability and did not scan the text for negations.

The KPSC team used those phrases and enriched lemma variants of the terms to address variation in describing the residential instability in the provider notes at their site. The team also added additional terms identified during their iterative process of prediction and chart review. The team did not assess temporality in the occurrence of residential instability but identified negation terms using ConTextNLP and terms describing the residential instability for someone other than the patient (eg, information on family members). This process resulted in 230 unique patterns constructed in spaCy.

The KPMAS team used a different approach to develop linguistic patterns. They used the Scikit-learn’s Term Frequency-Inverse Document Frequency (TF-IDF) Vectorizer feature extraction tool in their training data set.^31^ The tool extracted unigram (1-word) and 4-g (4-word) sequences with each unigram or 4-g sequence having an assigned TF-IDF score, calculated by using the product of term frequency and inverse document frequency and changing the weighting of term frequency to a logarithmic scale. In the end, all terms received a score between 0 and 1.

We then calculated the TF-IDF scores for each unigram and 4-g among provider notes originating from individuals with a positive residential instability label and the notes originating from all individuals and then reported a score difference between the 2 groups of notes for each unigram or 4-g. Arranging the terms by score difference in descending order we selected score differences ≥0 for manual annotation.

We further processed the unigram terms and 4-g phrases in 2 phases. First, we selected all unigrams based on their relevance to residential instability. We halted the unigram processing when 200 consecutive words were deemed irrelevant, ensuring a standardized and data-driven stopping point. Next, we utilized these unigram terms to limit the review of 4-g phrases and filtered the 4-g phrases containing 1 or more selected unigram terms.

To develop the linguistics patterns addressing residential instability, we imported the filtered 4-g phrases to Microsoft Excel for review by manual annotators.^32^ The annotators gave a binary label to each phrase to keep (1) or discard (0). We used the 4-g phrases that annotators labeled as keep to generate 2-component patterns with a starting and an ending component; requiring at least 1 component to remain specific to the residential instability, then excluded the duplication among generated patterns. We displayed patterns as component 1→ component 2 (eg, housing → assistance), where “→” represented any number of words, characters, or spaces between 2 components of interest. We did not assess temporality for the occurrence of residential instability but identified negation and false-positive matches using PhraseMatcher (a spaCy function). We reviewed 10 000 TF-IDF ranked phrases, developed 100 patterns, and implemented them in the PhraseMatcher NLP model. Table 1 presents a summary of different approaches across study sites for the gold standard and feature development.

**Table 1.** Approach for gold standard and feature development across study sites

	JHHS	KPMAS	KPSC
NLP validation			
Gold standard method	Social needs questionnaire	Social needs questionnaire	Social needs ICD codes & manual annotation
Training and validation data sets			
Patients/responses (count)	3572^a^	8197^a^	300^b^
	(1786+/1786−)	(833+,7364−)	(150+/150−)
Clinical notes (count)	299 307	78 825	9575
Note type	All note types; lab results, radiology, and pathology reports excluded	Case management, complex care program, family practice, internal medicine, psychotherapy, and utilization management departments	All note types; discharge instructional/administrative notes excluded
Breakdown of training and validation data sets	Randomly selected 50% of the sample for the training data set and reserved the remaining subset for a hold-out validation data set	Randomly selected 80% of the sample for the training data set and reserved the remaining subset for a hold-out validation data set	Randomly split the sample into 5 subsets (each with 30+/30− patients). Used 4 of the subsets for the training data set and the fifth set as a hold-out validation data set
Feature development			
	Hand-crafted linguistic patterns	TF-IDF Vectorizer feature extraction	Hand-crafted linguistic patterns

^a^ Total number of patients and those with positive and negative responses to residential instability questions in the social needs questionnaire included in the training and validation data sets. JHHS site then randomly selected 50% of the total sample to develop the training data set and reserved the remaining subset for evaluation of model over-fitting in a hold-out validation data set. KPMAS randomly selected 80% of the total survey data set to develop the training data set and reserved 20% of the total survey data set, of which 833 were positive and 7364 were negative for residential instability response, for evaluation of model over-fitting in a hold-out validation data set.

^b^ KPSC randomly split the total sample into 5 subsets, each set containing 30 patients from the positive and 30 patients from the negative residential instability groups. We used 4 of the subsets for the iterative adaption of the NLP algorithm and the fifth set of 30 positive and 30 negative patients for the final evaluation of the model over-fitting as a hold-out validation data set.

JHHS: Johns Hopkins Health System, KPMAS: Kaiser Permanente Mid-Atlantic States, KPSC: KP Southern California, NLP: Natural Language Processing, TF-IDF: Term Frequency-Inverse Document Frequency (TF-IDF).

### Provider note processing

The team at each study site performed preprocessing on the extracted provider notes, including (1) cleaning special and nonword or digital characters (eg, removing the *dot-phrase* or segments with extraneous formatting characters that may interfere with model performance), (2) spell checking and correction for mistyped, misspelled, or concatenated words detected during the NLP development process in previous studies, (3) sentence separation, and (4) tokenization (ie, segmenting text into linguistic units such as words and punctuation).^33^^,^^34^ We did not use any section identification, left the note sections undivided, and searched the entire provider note for NLP model development as our clinical experts recommended not to do so. The rationale was that residential instability can be identified in any part of the notes and focusing on specific sections (eg, social history) might result in missing some information.

### NLP model training

We applied spaCy’s open-source natural language processor to process and interpret unstructured provider notes.^35^ Using the EntityRuler module of the spaCy 2.3 Python toolkit, we created a rule-based NLP system made up of the expert-developed patterns that, if present, would represent residential instability. Our patterns included word ‘lemmas’ and base forms to account for morphological variations (eg, singular and plural forms) as well as substitutions of different prepositions (eg, about and for), and synonym words (eg, house, apartment, and home). We utilized SpaCy’s PhraseMatcher function to efficiently identify phrases indicating residential instability using the developed patterns. The process included searching each sentence for patterns addressing residential instability patterns. We did not search for historical terms or historic dates of residential instability. We considered the identification of negated patterns and patterns describing residential instability for someone other than the patient or the actual situation (false positive) as the absence of residential instability for the patient. We revised and optimized the patterns through an iterative application of the natural language processor within the training data set. We completed pattern revision and optimization before model implementation on the validation data set. Consequently, the validation data set did not influence the pattern generation and revision.

### NLP model prediction evaluation

If after removing negation matches and false-positive patterns at least 1 positive match remained in the note, we assigned a final prediction label of 1 to that provider note. If zero positive matches remained, we assigned a final prediction label of 0 to the provider note.

To assess the performance of the NLP algorithms at the patient level, the JHHS and KPMAS sites used housing questionnaires (eg, YCLS Survey at KPMAS). Thus, we linked all provider notes for each patient to their corresponding questionnaire and aggregated their scores. We assigned a questionnaire-level prediction score of 1 if the aggregate score from all provider notes was ≥1 and 0 if the aggregate score was 0. We compared the final questionnaire-level response predictions to the responses provided by patients to the questionnaire and assessed overall positive predictive value (PPV) (precision), sensitivity (recall), and specificity. The KPSC site used its validation data set developed through chart review. Therefore, they identified a patient as positive for residential instability if they identified ≥1 positive match in ≥1 note.

## RESULTS

The frequency of residential instability identified in the limited assessment of patients varied across study sites; 1786 (3.8%) patients at the JHHS and 2905 (11.3%) patients at the KPMAS had a positive response to the questionnaires and were considered residentially unstable. The KPSC site randomly selected 150 hospitalized or ED patients without residential instability diagnosis or homeless checklist and 150 with residential instability diagnosis or homeless checklist, 138 of those were identified as residentially unstable after the chart review process.

The demographic characteristics of the study populations were slightly different across different sites. Patients with residential instability at the KPSC were younger than those at JHHS and KPMAS (52.9% under the age of 45 at KPSC vs. 32% at JHHS and 29.2% at KPMAS) and were more male (63.8% at KPSC vs. 37.3% at JHHS and 36.3% at KPMAS). In terms of race/ethnicity, notable differences were identified among the 3 study sites; 49.5% and 56.5% of patients with residential instability were non-Hispanic blacks at JHHS and KPMAS sites. At KPSC, however, non-Hispanic whites had the highest number of residential instability (34.1%) followed by Hispanics (32.6%). In terms of insurance information, the majority of JHHS patients did not have data available on their insurance coverage (86.8% were listed as other insurance which also included self-pay). At the KPMAS site, the majority of patients (51.1%) and those with residential instability (37.7%) were Medicare patients. Among all patients, those with a standard Health Maintenance Organization (HMO) (22.4%) and Medicaid (17.1%) were second and third, while among patients with residential instability Medicaid patients ranked as second (30.2%), followed by those with a standard HMO (22.0%). Among KPSC patients, the majority of patients (42.0%) and those with residential instability (74.5%) had other insurance coverage with the majority of them being non-KP members. Table 2 presents the characteristics of the study population at each study site.

**Table 2.** Characteristics of the study population at each study site

	JHHS		KPMAS		KPSC	
	Total	Residential instability	Total	Residential instability	Total	Residential instability
Study population, *n* (%)						
	47 440 (100)^a^	1786 (3.8)	25 727 (100)^b^	2905 (11.3)	300^c^	138
Age, *n* (col%)						
18–34	4592 (9.7)	283 (15.8)	3612 (14.0)	505 (17.4)	97 (32.3)	39 (28.3)
35–44	3303 (7.0)	290 (16.2)	1858 (7.2)	342 (11.8)	57 (19.0)	34 (24.6)
45–64	12 526 (26.4)	747 (41.8)	8404 (32.7)	1217 (41.9)	95 (31.7)	52 (37.8)
65–74	8732 (18.4)	186 (10.4%)	5634 (21.9)	467 (16.1)	27 (9.0)	11 (8.0)
≥75	12 996 (27.4)	109 (6.1%)	6219 (24.2)	374 (12.8)	24 (8.0)	2 (1.5)
Gender, *n* (col%)						
Male	25 425 (53.6)	667 (37.3)	10 204 (39.7)	1053 (36.3)	156 (52.0)	88 (63.8)
Female	22 013 (46.4)	1119 (62.6)	15 523 (60.3)	1852 (63.7)	144 (48.0)	50 (36.2)
Race/Ethnicity, *n* (col%)						
Hispanic	6 (0.01)	1 (0.05)	2010 (7.8)	222 (7.7)	112 (37.3)	45 (32.6)
Non-Hispanic Black	15 102 (31.8)	844 (49.5)	12 345 (48.0)	1641 (56.5)	42 (14.0)	24 (17.4)
Non-Hispanic White	26 899 (56.7)	801 (44.8)	8547 (33.2)	820 (28.2)	96 (32.0)	47 (34.1)
Asian/Pacific-Islander	1802 (3.8)	12 (0.7)	2244 (8.7)	193 (6.6)	21 (7.0)	2 (1.5)
Other/unknown	3483 (7.3)	115 (6.4)	581 (2.3)	29 (1.0)	29 (9.7)	20 (14.5)
Insurance, *n* (col%)						
Medicaid	31 (0.1)	3 (0.2)	4391 (17.1)	877 (30.2)	21 (7.0)	8 (5.8)
Medicare	0 (0)	0 (0)	13 154 (51.1)	1,095 (37.7)	45 (15.0)	8 (5.8)
Deductible	702 (1.5)	12 (0.7)	2114 (8.2)	236 (8.1)	19 (6.3)	3 (2.2)
Standard HMO	236 (0.5%)	11 (0.6)	5756 (22.4)	640 (22.0)	89 (29.7)	16 (11.6)
Other	41 180 (86.8)^d^	1589 (88.9)	312 (1.2)	57 (2.0)	126 (42.0)^e^	103 (74.5)^f^

^a^ The JHHS site assessed over 30 EHR questionnaires and flowsheets addressing residential instability and identified 5 relevant ones. Between July 2016 and June 2020 we identified, 1786 patients with positive response and 45 654 patients with negative response to residential instability questions.

^b^ The KPMAS site extracted the YCLS survey data from the EHR. Between March 2017 and June 2020 we identified a total of 40 372 YCLS survey responses completed by 25 727 KPMAS adult members, 2905 of whom indicated residential instability. We used YCLS survey responses to generate a binary label of a patient’s needs related to residential instability. We assigned a positive label (1) to patients with a survey response indicating an unmet housing need and a negative label (0) to patients with a survey response indicating no current housing need.

^c^ KPSC site randomly selected 150 hospitalized or ED patients between January 2016 and December 2019 with residential instability diagnosis or homeless checklist and 150 without residential instability diagnosis or homeless checklist. Positive cases (138 patients) were those identified as residentially unstable during the chart review process.

^d^ Including self-pay.

^e^ 124 of 126 are non-KP members.

^f^ 102 of 103 are non-KP member.

EHR: Electronic Health Record, HMO: Health Maintenance Organization, JHHS: Johns Hopkins Health System, KPMAS: Kaiser Permanente Mid-Atlantic States, KPSC: KP Southern California, YCLS: Your Current Life Situation.

To assess the performance of the NLP algorithm at JHHS, we included 1786 patients with and the same number without residential instability and a total of 299 307 provider notes for those patients (Table 1). Notes originated from 51 provider types and 99 clinical departments of interest. At KPMAS, we included 833 patients with and 7364 without residential instability and a total of 78,825 provider notes for those patients, originating from 6 clinical departments of interest. At KPSC, we included 150 patients with and the same number without residential instability and a total number of 9575 notes for those patients. Note types varied across the 3 sites with JHHS and KPSC including almost all note types and KPMAS including selected ones. Our NLP system reviewed all the included clinical notes at each study site. The NLP algorithm performed relatively well across the 3 sites with PPV (precision) of 0.73, 0.45, and 1.00, sensitivity (recall) of 0.85, 0.68, and 0.96, and specificity of 0.69, 0.89, and 1.00 for JHHS, KPMAS, and KPSC, respectively.

## DISCUSSION

The increase in the number of available NLP systems and the need to unlock rich free-text notes for clinical information highlights the importance of developing efficient systems to process large corpora of free text.^24^^,^^36^^,^^37^ Such systems must be adaptable from 1 healthcare system to another to increase the widespread use of this advanced health information technology tool in the healthcare sector. In the current project, we assessed the generalizability of a rule-based NLP system to extract markers of residential instability from Epic-based EHRs in 3 different healthcare systems. Thus, we made modifications to the base NLP system developed at the JHHS site to address data availability and the unique digital workflow of each healthcare system.

Most NLP systems are designed to extract clinical information stated in the notes using generally accepted common terminologies for documenting clinical issues (eg, explicit documentation of drug and alcohol use).^36^^,^^38–40^ The main difference between such information and information related to the social needs of a patient is that social needs are often not explicitly stated in the clinical notes, but often this information can be inferred from provider comments describing the patients living situation or environment. For instance, from the statement “patient sleeps on her friend’s couch,” it can be indirectly inferred that the patient has housing insecurity.^22^ The inference also requires the processing of highly ambiguous colloquial words. For instance, to process the sentence “patient has to stay at the hospital overnight because he has no place to go after the procedure” requires identification of everyday words, tasks, and roles, in addition to inference, capabilities to arrive at the (correct) conclusion that the patient is homeless.^22^

Our results were similar to other studies using state-of-the-art NLP systems to identify social needs in free-text provider notes. For instance, Conway et al^22^ tested the performance of Moonstone, a new, highly configurable rule-based clinical NLP system for extraction of information requiring inferencing from clinical notes derived from the Veterans Health Administration. Their system achieved a precision of 0.66 (comparable with the precision of 0.45–0.96 across 3 sites in our study) and a sensitivity of 0.87 (comparable with the sensitivity of 0.68–0.96 across 3 sites in our study) for phrases related to homeless and marginally housed. Navathe et al^13^ utilized MTERMS, an NLP system validated for identifying clinical terms within medical record text to extract social factor information from physician notes. They customized and developed the MTERMS NLP system on a randomized 500 annotated physician note training set and tested the diagnostic characteristics. After development, they validated the system by studying the diagnostic characteristics of the system versus a gold standard manual review of a new set of randomized 600 physician notes. They achieved a precision of 1.0 and a sensitivity of 0.66 for housing instability. Gundlapalli et al^41^ developed an open-source NLP tool (Automated Retrieval Console v2.0 [ARC]) and trained the tool using a human-reviewed reference standard corpus of clinical documents of Veterans with evidence of homelessness and those without. The best-performing model based on document level workflow performed well on a test set (Precision 94%, Recall 97%, and F-Measure 96). The human review noted a precision of 70% for these flags. Gundlapalli et al^42^ also used the V3NLP Framework, a UIMA^43^ based set of tools, annotation label guidelines, annotators, readers, and writers designed to aid NLP developers to build out applications. The framework evolved initially from other widely used NLP systems such as CTAKEs.^40^ The framework detected instances of lexical terms with a precision value of 77% for extracting relevant concepts.

Other notable mentions include a rule-based algorithm developed by Hollister et al to extract social needs data from racial/ethnic minority adult patients in BioVU, the Vanderbilt University Medical Center biorepository linked to deidentified EHRs. They compared the social need data extracted from a manual review of 50 randomly selected records to data produced by the algorithm, resulting in a precision of 33.3% for patients with homelessness.^44^ In another study, Dorr et al^23^ extracted the phenotypic profiles for 4 key psychosocial vital signs including housing insecurity or homelessness from EHR data. They used lexical associations expanded by expert input, then, for each psychosocial vital sign, and manually reviewed the retrieved charts. Their system achieved a precision of >0.90 in all psychosocial vital signs except for social isolation. While these well-developed NLP systems have presented variable levels of success in extracting social needs information from EHR free-text notes, all the attempts were limited to the isolated healthcare system. To the best of our knowledge, our study is one of the first attempts to assess the performance of a rule-based NLP system across different healthcare systems with different data availability and digital workflows. The generalizability of the NLP systems to be applied to different healthcare systems is an important topic of study. Our findings add to the current literature by implementing and comparing the performance of an NLP system across different sites. The different approaches taken by each study site demonstrated many ways to develop and implement a clinical NLP system.

In our study, the precision and recall for the NLP algorithm varied across the 3 sites. The NLP system performed the best using the EHR data at the KPSC site (ie, the precision of 1.00 and recall of 0.96). The better performance may be due to a more accurate gold standard and validation data set for the assessment of the performance of the NLP system. The team also performed adjustments in the NLP system including enriching the base phrases with lemma variants of the terms to address variation in describing the residential instability in the provider notes at their site. Moreover, they added additional terms identified during their iterative process of prediction and chart review. This process helped to adapt the base NLP algorithm to the KPSC site. But might have resulted in an NLP system with high specificity which might not perform at the same level in other sites. Also, the small sample size of the validation data set at the KPSC site should be taken into concentration. In contrast, using questionnaires for the development of the validation data set at JHHS and KPMAS resulted in lower performance. The questionnaires often lack specific questions when it comes to ambiguous social needs such as housing insecurity when there is a lack of consensus among providers on how to define and identify such social needs. An error analysis across the 3 sites revealed the false positive instances as the common source of error; the false positive instances were either due to negation or empty values (eg, homelessness: no, homeless: NA or [empty values in free text], and housing instability: [empty space]). KPMAS site identified and addressed these false positives using the negative PhraseMatcher. Another common source was false negatives due to specific names for shelters and other support facilities (eg, referred to [specific name for a shelter]). While assessing type I and type II errors through reviewing each potential match error and classifying/quantifying them were not feasible, given the volume of training data sets across the sites, the evaluation of accuracy using precision and recall provided sufficient information for this pilot study to compare the performance of the NLP algorithms across 3 sites.

We also experimented with different approaches to feature development and used both manual lexicon curation and semiautomated lexicon creation.^24^ As the first approach at the JHHS site, we used a manual lexicon curation approach and developed hand-crafted linguistic patterns after reviewing several medical terminologies and the description of residential instability in public health surveys and instruments, conducting a literature review of past studies, and utilizing the results of manual annotation.^42^ We used those phrases at the KPSC site and included lemma variants and additional terms that were identified through chart review. We used a semiautomated lexicon creation approach and developed a TF-IDF Vectorizer feature extraction tool at the KPMAS site. Similar to other established semiautomated approaches such as word2vec,^45^ this data-driven approach helped our team to automatically extract a feature describing residential instability from thousands of clinical notes. Unlike Bejan et al’s study,^45^ we did not use any relevant seed keywords. We designed the method on the premise that the best candidate words to describe the residential instability are the ones that occur in provider notes originating from individuals with a positive residential instability label. We manually assessed the top-ranked words generated by this method and included the highly relevant ones in the residential instability query.^45^ The word2vec experiments in Bejan et al’s study^45^ resulted in a higher precision value (at the 50th ranked word, the precision of 0.80 and 0.82 for context size of 5 and 15 words, respectively) compared to our approach. As stated earlier the better performance may be due to a more accurate gold standard and validation data set rather than the limitations of the semiautomated lexicon creation approach. Selecting different approaches to feature development helped us to assess the performance of the NLP algorithm in different healthcare systems and to address variations in the documentation of the residential instability across the systems.

Overall differences in the gold standard development and NLP methods often lead to different model performance ranges across healthcare systems. However, our different approaches to various tasks in this process such as selecting the note types, developing the features, and creating the validation data sets across study sites and settings, were complementary and helped to provide a comprehensive assessment of the NLP algorithms.

Several challenges are associated with using clinical notes for NLP purposes. A challenge is that the EHR clinical notes often are highly templated (ie, semistructured), including checkboxes and structured question and answer templates. For instance, homelessness can be represented in clinical notes in different ways (“patient is not homeless,” “homeless: 1,” “homeless: yes”).^22^^,^^46^ Therefore, automatic distinction between free text, structured and semistructured areas of the clinical note is an existing challenge in this domain.^47^ Moreover, clinical notes contain several idiosyncratic abbreviations and truncations, missing function words, ambiguity, and misspellings. To address this challenge, our team performed pre-processing on the extracted provider notes to clean special and nonword or digital characters, performed spell checking and correction for mistyped misspelled, or concatenated words, and conducted sentence separation and tokenization (ie, segmenting text into linguistic units such as words and punctuation).^33^ Future research should explore the creation of special-purpose NLP tools for the identification of semistructured data and narrative text, and preprocessing text, especially for the identification of social needs in the EHR’s free text.^22^ Finally, there are different NLP techniques for the review of clinical notes. These techniques range from the linguistically oriented rule-based NLP systems made up of expert-developed patterns, similar to the one we used in this study, to machine learning techniques such as modern neural network-based machine learning.^48^ The rule-based techniques use a much smaller data set as opposed to the annotated data necessary to both train and evaluate a machine learning algorithm. They are also less opaque than machine learning-based NLP algorithms and the reasons for a particular classification decision can be articulated.^22^ Therefore, they help to develop NLP algorithms with a higher level of adaptability and without challenges related to data sharing across healthcare systems.

Our study had other limitations. The prevalence estimation of residential instability may underestimate the magnitude of the problem. The true prevalence estimation requires a significant time-intensive manual assessment effort, which was out of the scope of our study.^45^ Aggregating several notes created over some time and linking them to a single survey response to evaluate the performance of the NLP algorithms limited our ability to assess the temporality of the identified social needs. To address this issue, it would be necessary to assess the performance on a note-by-note basis. Since residential stability is a long-term social need, future studies should also assess the temporality of residential stability and similar social needs. We tested the performance of the baseline JHHS NLP algorithms in the KPSC site, which resulted in a large list of false-positive phrases. KPMAS site used an early version of the JHHS NLP model but was not able to apply the final iteration of this NLP model given time and resource constraints. To truly assess the generalizability of the NLP system, we needed to evaluate the performance of the modified NLP algorithms in each of the healthcare systems, which was beyond the focus of this study and available resources. We tested our NLP system in 3 integrated healthcare delivery systems, physician documentation of social needs and risk factors in clinical notes vary among different healthcare systems and our study sites may not be representative of all types of documentation styles and preferences. Developing rule-based NLP systems requires deep knowledge of the domain and is time-consuming to generate complex rules to address all challenges related to the complexity of describing social needs and addressing negations and false-positive patterns. Furthermore, the rule-based approach requires a skilled linguist expert to manually craft and enhance each NLP rule, which might result in a complex system with some rules contradicting others. Such complexity might limit the development and use of the system to larger healthcare systems with a well-developed informatics infrastructure.

## CONCLUSION

Despite the limitations, the promising performance of our NLP system to identify residential instability in 3 different healthcare systems suggests the algorithm can be adapted across comparable healthcare systems and EHR settings. The relatively high sensitivity and specificity demonstrate the algorithm’s validity. The development of adaptable NLP systems with promising performance will enhance the value of EHRs to identify at-risk patients across different healthcare systems, improve patient care and outcomes, and mitigate socioeconomic disparities across individuals and communities.

## FUNDING

This work was supported by the Johns Hopkins Institute for Clinical and Translational Research (ICTR) which is funded in part by Grant Number UL1 TR003098 from the National Center for Advancing Translational Sciences (NCATS) a component of the National Institutes of Health (NIH), and NIH Roadmap for Medical Research. Its contents are solely the responsibility of the authors and do not necessarily represent the official view of the Johns Hopkins ICTR, NCATS, or NIH.

## AUTHOR CONTRIBUTIONS

Study concept and design: EH, CN, and DR. NLP algorithm development and testing: MR, FX, CR, and MA-N. Interpretation of Results: EH, CN, and DR. Drafting of the manuscript: EH, MR, CN, FX, AP, LJL, CR, MA-N, and DR. Critical revision of the manuscript for important intellectual content: EH, MR, CN, FX, CR, MA-N, HK, and DR. Chart review and manual annotation: AP and LJL, Administrative, technical, and material support: EH, CN, HK, JPW, and DR. Study supervision: EH, CN, and DR.

## SUPPLEMENTARY MATERIAL

Supplementary material is available at *JAMIA Open* online.

## DATA AVAILABILITY

The data underlying this article were extracted from the electronic health records at 3 study sites and cannot be shared publicly for the privacy of individuals that participated in the study. 

## CONFLICT OF INTEREST

None declared.

## Supplementary Material

ooac006_Supplementary_Data
