# Supplementary material for: Development and assessment of a natural language processing model to identify residential instability in electronic health records’ unstructured data: a comparison of 3 integrated healthcare delivery systems
Source: JAMIA Open. 2022 Feb 16;5(1):ooac006. doi: 10.1093/jamiaopen/ooac006 (PMC8867582; doi:10.1093/jamiaopen/ooac006)
Supplement: ooac006_Supplementary_Data [file ooac006_Supplementary_Data.docx]

| **Supplementary Table 1. Categories of Residential Instability and Example Phrases In Free Text Notes at Three Study Sites** | | |
| --- | --- | --- |
| **Category** | **Definition** | **Examples** |
| Homelessness | Refers to phrases used by clinicians, social workers, or staff members to indicate a current homelessness status or lack of housing OR | patient has nowhere to stay (JHHS)  homelessness 🡪 crisis (KPMAS)^1^  medical social worker initial assessment: homeless (KPSC)  patient is homeless off and on for approximately 1 year (KPSC)  patient has been residing on the streets for several months (KPSC) |
|  | Indicates when a clinician, social worker or staff member addresses the homelessness status of a patient through various interventions | Coordinated housing services (JHHS)  homeless 🡪 assist (KPMAS)^1^  housing 🡪 assistance (KPMAS)^1^  patient is interested in looking into shelters (KPSC) |
| Housing Insecurity | Indicates challenges with paying rent or spending the bulk of household income on housing that a patient has faced OR | patient occupied his house without rent payment (JHHS)  currently 🡪 no rent (KPMAS)^1^ |
|  | Indicates challenges captured in the clinical notes with moving frequently or staying with relative and friends OR | patient has difficulty affording a stable place (JHHS) |
|  | Indicates challenges with overcrowding or other challenges patient has faced with the poor quality of their place of residence | patient lives with several family members in a studio (JHHS) |
| ^1^At KPMAS patterns were displayed as component 1🡪 component 2, where “🡪” represents any number of words, characters, or spaces between two components of interest.  JHHS: Johns Hopkins Health System, KPMAS: Kaiser Permanente Mid-Atlantic States, KPSC: KP Southern California | | |

| **Supplementary Table 2. Logic to Classify Residential Instability Responses in Surveys and Questionnaires** | |
| --- | --- |
| **Question Assessing Status of Residential Instability** | **Responses Indicating Presence of Residential Instability** |
| **Johns Hopkins Health System** | |
| **Question** | **Answer** |
| Q1: Homeless: is the patient homeless? | Yes/No |
| Q2: Need help with finding housing? | Yes/No |
| **Kaiser Permanente Mid-Atlantic States** | |
| **YCLS Question** | **Answer** |
| Q1: Which of the following best describes your current living situation? (Select ONE only) | 5: Temporarily staying with a relative or friend  6: Temporarily staying in a shelter or homeless |
| Q2: Do you have any concerns about your current living situation, like housing conditions, safety, and costs? | 1: Condition of housing  2: Feeling safe  3: Ability to pay for housing or utilities  4: Lack of more permanent housing |
| Q3: In the past 3 months, did you have trouble paying for any of the following? (Select ALL that apply) | 2: Housing  3: Heat and electricity |
| Q8: Which of the following would you like to receive help with at this time? (Select ALL that apply) | 2: Housing  4: Utilities (heat, electricity, water, etc.) |
| YCLS: Your Current Life Situation | |
